# Social skills training with a tabletop role-playing game, before and during the pandemic of 2020: in-person and online group sessions

**DOI:** 10.3389/fpsyt.2023.1276757

**Published:** 2024-01-05

**Authors:** Germano Henning, Reinaldo Rodrigo de Oliveira, Marcus Túlio Pereira de Andrade, Renato Villela Gallo, Raissa Roberti Benevides, Rodrigo Antonio Fuga Gomes, Lucas Eiji Kong Fukue, Arthur Vaciloto Lima, Maria Beatriz Baggio Z. N. de Oliveira, Daniel Amorim Medeiros de Oliveira, Morgana Werpp, Lucas Moraes, Francisco Lotufo Neto

**Affiliations:** ^1^Institute of Psychology, University of São Paulo, São Paulo, Brazil; ^2^Department of Psychiatry, University of São Paulo, São Paulo, Brazil; ^3^Department of Neuroscience, Pontifical Catholic University of Paraná, Curitiba, Brazil; ^4^Department of General Psychology and Behavior Analysis, State University of Londrina, Londrina, Brazil; ^5^Department of Psychology, Pontifical Catholic University of São Paulo, São Paulo, Brazil; ^6^Department of Psychology, Pontifical Catholic University of Rio Grande do Sul, Porto Alegre, Brazil; ^7^Department of Psychology, Nove de Julho University, São Paulo, Brazil

**Keywords:** social skills training, tabletop role-playing game, online therapy, group psychotherapy, behavior analysis, pandemics

## Abstract

**Background:**

The area of social skills is broad, in theory and in practice. For social skills training, various clinical practices have been applied in group sessions, as have motivational resources such as role-playing games (RPGs). In recent years, the need arose to assess the clinical impact of the pandemic. The objective of this study was to determine the impact that the pandemic has had on in-person and online social skills training.

**Methods:**

We evaluated six subjects with autism spectrum disorder, with or without another, similar disorder, each of whom attended a total of 12 two-hour RPG sessions over a 12-month period. The original (Portuguese-language) version of the Social Skills Inventory for Adolescents was applied at three different time points (pre-, mid-, and post-intervention).

**Results:**

After six in-person tabletop RPG sessions, there was an increase in the mean frequency scores and a decrease in the mean difficulty scores. However, during the pandemic, the remaining six sessions were conducted online and the effect was the opposite.

**Conclusion:**

Our data indicate that there is a need for further studies assessing social skills training in online contexts.

## 1 Introduction

The area of social skills is relevant in clinical and social contexts, not only in theory but also in application. Social skills are known to have an effect on quality of life and on psychiatric symptoms. Various studies, involving individuals with or without psychiatric disorders, have shown that the clinical picture is better among those with social skills that are more well developed (1, 2). Some instruments—such as the World Health Organization Quality of Life assessment, the 45-item Outcome Questionnaire, the Child Behavior Checklist, the Group Climate Questionnaire, and the Parenting Style Inventory—assess not only clinical conditions and psychiatric symptoms but also interpersonal relationships, romantic relationships, interactions with parents, and other social aspects. Although the social skills that an individual possesses are not the only factor that determines the outcome of a social interaction, they are certainly relevant.

Because social skills constitute an important criterion for assessing well-being or mental health, their assessment is also a common step in psychological treatments for various clinical conditions, including anxiety, depression, and schizophrenia, as well as diagnostic criteria such as delinquency (1–4). Social strategies and social skills training are used in fields other than that of mental health. They have been employed for interpersonal training in companies, self-awareness training, marriage counseling, a purpose-driven approach to cultivating relationships, training in sales techniques, and making friends (5, 6). Improving social skills can also facilitate social inclusion between different audiences, especially between autistic and neurotypical individuals, between whom there can be a failure of mutual empathy. According to Milton et al. (7), recognizing the “double empathy problem” is important for understanding and accepting autistic people when they present common, stereotypical behaviors such as echolalia. The authors stated that neurotypical and autistic people have difficulty understanding and sharing feelings with each other. Because autism spectrum disorder (ASD) is a neurodevelopmental characteristic, people with ASD have processing and communication styles that are different from those of neurotypical people. Although social skills training can promote the inclusion of autistic people in society, there is a need for educational programs targeting the general population in order to reduce the stigma that autistic people face, which can result in prejudice and discrimination (8). There is also some concern that traditional social skills training can actually do harm to individuals with ASD (9).

Throughout the theoretical studies, there was some difficulty in defining what exactly is skillful social behavior, because it depends on a context that is changing and it is difficult to predict the consequences of a given social skill exhibited in a given social context (2). Social behavior may be appropriate in one context and inappropriate in another. If we consider all of the variables to understand what is appropriate or inappropriate, it becomes more difficulty to define exactly what skillful social behavior is. Such variables include the general or specific culture of the locale, as well as the age, sex, socioeconomic status, and level of education of the individuals in the target group (2, 3).

Because there is no clear definition of the topography of what constitutes skillful social behavior, some authors, such as Linehan (10), under the influence of radical behaviorism, argue that the outcome constitutes the criterion. For that author, there are three types of outcomes: efficacy to achieve the objectives; efficacy in interpersonal relationships; and efficacy in attaining or maintaining self-respect.

In their Social Skills Training Manual, Del Prette and Del Prette (3) differentiated between two fundamental concepts: social skills and social competence. Those authors defined social skills as a construct that describes social behaviors valued in a given culture, with a high probability of favorable results for the individual, the group, and the community, resulting in socially competent performance in interpersonal tasks. According to some authors, possessing good social skills does not guarantee good performance in a natural context. That is why, unlike Caballo (2), and Del Prette and Del Prette (3) defined social competence as a construct employed to evaluate the performance (thoughts, feelings, and actions) of an individual on an interpersonal task that meets the objectives of the individual, as well as the demands of the situation and culture, and produces results that meet instrumental and ethical criteria.

After defining the concept of social skills, it is now important to define the domains of social skills. On the basis of a long, historical process of study and discussion, described by Caballo (2), and Del Prette and Del Prette (3), ten social skills domains (some with subdomains) have been defined: communication; civility; making and maintaining friendships; empathy; assertiveness; expressing solidarity; handling conflicts and solving interpersonal problems; expressing affection and intimacy (dating and sex); coordinating groups; and public speaking. Programs for training in those domains are found in interventions that can have different objectives, according to each specific demand. Various social skills training programs have been employed in fields such as couples therapy (5) and children’s stress (11), as well as therapy for panic disorder and agoraphobia (12). Among the numerous specific programs for social skills training are the Program for the Education and Enrichment of Relationship Skills (PEERS), developed at the University of California, Los Angeles (13); The Junior Detective Training Program (14); the Social Skills Training Autism—Frankfurt program (15); the Social Competence Intervention Program (16); and the Treatment and Education of Autistic and Related Communication Handicapped Children program (17).

Andreozzi (18) showed that, for social skills training programs to be efficacious, interventions must be planned so that they can be generalized and social behavior can be qualified through the use of scales and direct observations of behavior by a trained observer. Various techniques for social skills training have been described in the literature. Such techniques include the following (2, 12–17, 19, 20): traditional instruction; modeling for learning through observation; modeling for learning through successive reinforcement of the target behavior; behavioral rehearsal; verbal and video feedback; homework; cognitive restructuring; problem solving; relaxation; and group therapy.

One of the strategies used in the literature is the application of a behavioral assay, and one of the ways it can be applied is through games. Various authors have used tabletop role-playing games (TTRPGs) as tools in interventions, focusing on the development of a specific repertoire of intentional speech directed at others, with the aim of improving the quality of life of the participants (21). Katō (22) presented the results of two studies employing a TTRPG to promote social communication and efficacious leisure activity in a group of children and adolescents with ASD. In the first study, which involved four adolescents, the author analyzed the number of verbalizations directed at no one (i.e., talking to oneself), comparing it with that of those directed at someone. The analyses were performed in the first and last of 14 sessions. In that study, there was a statistically significant increase in the number of verbalizations directed at a person and a decrease in the number of those directed at no one. The second study involved a sample of 51 adolescents and was designed to determine the impact of five TTRPG sessions on the quality of life of the subjects. A quality of life questionnaire was applied before and after the intervention, and the data show that there was improvement in the quality of life of the participants, as evidenced by statements such as “After trying the tabletop RPG, I like to talk more than before.” The subscales of the quality of life questionnaire showed improvement mainly in the domains “quality of emotion” and “friends” (22).

Katō (22) discussed the characteristics of using a TTRPG as a tool for social skills training. First, leisure activities and hobbies are important to create bonds of friendship between children and young people with ASD. A simple conversation about a hobby facilitates interaction between the participants. In addition, tabletop role-playing works differently from colloquial conversation. The elements involved seem to facilitate interpersonal relationships among children and adolescents with ASD, such as rules and configuration of activities, clarity of objectives, common functions, and indirect communication through a character (an avatar). Furthermore, a TTRPG can rapidly improve quality of life in the population of individuals with ASD, even when no specific training in provided. Moreover, despite the stereotype that young people with ASD are not good at communicating or dislike group activities, leisure activities can improve social interactions if the setting allows spontaneous interactions. Therefore, the use of TTRPGs can help autistic people engage in playful activities, because, when well structured, with clear rules and adapted materials, games facilitate the development of social skills (23).

In their original context, TTRPGs were designed to promote playful activity. The simulation of a real scenario, such as a fight, rarely poses a threat to the participants, even with the recognition of a winner and a loser (22, 23). The opportunity to engage in playful activity and to practice such behaviors can result in the improvement of several cognitive aspects, such as creativity, logical imagination, reading acquisition, reasoning, and critical reflection (24, 25).

In TTRPGs, players represent characters, creating a narrative built collaboratively with other players. Typically, TTRPGs are cooperative, social games, although there are some that promote competition. The first book about an TTRPG was published in 1974, with the launch of the game Dungeons & Dragons (26), which was inspired by board games and war strategy, a known game genre. Traditionally, a game of Dungeons & Dragons is played at a table, around which all of the players sit. One has the function of dungeon master or game master (story narrator), and the rest are the players. Each player creates a unique character, and the game progresses in accordance with the course of the adventures planned by the master. The authors of Dungeons & Dragons set the game in a fantasy universe, inspired by various mythologies that have influenced the culture of children and adolescents.

Dungeons & Dragons is set within the epic fantasy genre. In addition to the possibilities for creating characters from different universes, the game allows the points earned in each adventure to accumulate. As each level is reached, the character also unlocks new skills, becoming a stronger, more versatile character for dealing with the various scenarios within the game. The dungeon master can use miniatures of the characters and maps of the area to be explored, thus facilitating understanding of the game scenario. Although the possibilities for character activities are almost endless, there is a complex system of rules that help the dungeon master determine whether a given character action is possible or not. All of this fictional and imaginative context has influenced many young people around the world. Garcia (24) stated that, even 40 years after its publication and in its fifth edition, Dungeons & Dragons continues to influence young people around the world through board and electronic variants of the game. Thus, it continues to inspire many young people through pop culture.

Because of their motivational aspects and versatility of application, RPGs have has been used in various areas, including education (27), mental health (21, 22, 27–31), and the analysis of their impact on pop culture (24, 25).

In the study conducted by Katō (22), the aim was not to intervene directly in various categories of social skills, as it was in those conducted by Caballo (2), and Del Prette and Del Prette (3); rather, it was to intervene in verbalizations directed at no one and intentional speech directed at others. Katō (22) compared the frequency of target behaviors between the initial and final sessions (i.e., pre- and post-intervention).

In 2015, Henning and Andrade (28) conducted a study involving behavioral intervention via group therapy in adolescents with ASD in Brazil, using RPG reinforcers to perform a direct intervention targeting the various domains of social skills, with the objective of developing skills in the categories proposed by Del Prette and Del Prette (3). Since then, that practice has been ever more widely implemented, with the use of RPG reinforcers and gamification in clinical and nonclinical contexts, with a focus on training individuals to generalize the skills acquired (29–31). To our knowledge, there have been no studies specifically assessing the categories of social skills, as described by Del Prette and Del Prette (3), and adapting a TTRPG to strengthen those social skills.

During the COVID-19 pandemic, psychotherapy had to adapt to conform to the new online format, emerging as a novel and widely adopted modality in mental health treatment (32). It is noteworthy that there was a significant increase in cases of anxiety and depression, particularly among adolescents, during the pandemic, underscoring the essential role of psychotherapy, especially in this demographic (33, 34). There was also an increase in the number of patient complaints related to social isolation (35).

The objective of the present study was to determine the impact of a TTRPG-based intervention in adolescents with ASD, analyzing the therapeutic process in detail, from one session to another, considering the group session environment, as well as evaluating the impact on participant quality of life and the interaction between the participants and their parents. A secondary objective was to determine the impact that the coronavirus disease 2019 pandemic had on the therapeutic process.

## 2 Method

### 2.1 Participants

The study sample comprised six adolescents between 13 and 17 years of age, all of whom had confirmed psychiatric diagnoses of ASD, based on the criteria defined in the Diagnostic and Statistical Manual of Mental Disorders, Fifth Edition. Because it involved research with humans, especially because those humans were adolescents, the study, including the necessary adaptations due to the pandemic, was periodically approved by the Research Ethics Committee of the University of São Paulo Institute of Psychology throughout the study period (Reference no. 40394620.4.0000.0068). The study was conducted in accordance with the ethical principles of the Declaration of Helsinki.

The inclusion criteria were having an interest in elements of pop culture and complaining of a deficit of social skills. Individuals who had engaged in self-harming behavior or had attempted to harm others were excluded, as were those who had moderate or severe autism, because their difficulties in speech and abstraction would have impaired their ability to participate.

The therapists who participated in the research were graduate students in psychology with 3–9 years of experience in behavioral therapy. All of those therapists followed the research from the beginning and participated in the development of this intervention, which was aimed at social skills training through the use of a TTRPG.

Throughout the study period, all of the sessions were group sessions, with four participants in one group (one of the participants in that group was older, outside the IHSA age range, and was therefore not included in the analysis) and three participants in the other. In each group, there were two therapists. The makeup of each group, in terms of the individual therapists and participants, did not change between sessions or over the course of the study period.

### 2.2 Instruments

For the selection of individuals for the groups, we used a simple, self-report questionnaire on interests that includes questions regarding attitudes about cartoons, YouTube, video games, social networks, relationships, graphic novels, sports, and e-sports. We also applied the *Inventário de Habilidades Sociais para Adolescentes* (IHSA, Social Skills Inventory for Adolescents), devised by Del Prette and Del Prette (36), which is a self-report questionnaire composed of 38 items, divided among six subscales: empathy; self-control; civility; assertiveness; affective perspective; and social resourcefulness. All of the items are scored for frequency and difficulty. On any given item, the social behavior in question is considered to be more appropriate if the score for frequency is high and the score for difficulty is low. For each item, the questionnaire calls for the participants to remember what they were supposed to do in the last ten situations and whether or not they did it. The IHSA was applied at three different time points (pre-, mid-, and post-intervention), whereas the instruments described below were applied in every session.

To assess the progress made in the interactions among participants from session to session, we also used the Group Climate Questionnaire (GCQ), developed by the Outcome Questionnaire Measures group (37). The GCQ is a self-report inventory designed to collect information regarding the perceptions of the participants regarding the interpersonal climate within the group. The inventory consists of twelve items, for each of which the participant communicates how much they agree with a statement or whether an event occurred during the group session, using a 7-point Likert scale, ranging from *not at all* to *extremely*. The twelve items are divided among three subscales: engagement, avoidance, and conflict. Thus, the therapist can assess the therapeutic process for an individual over the course of the sessions. The CQC was applied at the end of each session.

Another instrument employed in the present study was the Clinical Global Impressions (CGI) scale, a tool used in clinical research and practice that allows researchers and clinicians to assess the severity of and change in the symptoms of disorders over time, tracking changes in the clinical picture during treatment (38). It comprises two blocks: severity and improvement. The first block assesses the symptoms at the time of evaluation, with response options ranging from 0 (not assessed) to 7 (among the most extremely ill). The second block assesses the change in the severity of symptoms since the last assessment, with response options ranging from 1 (very much improved) to 7 (very much worse). For the purposes of the present study, the CGI scale was used in order to rate what the parents or guardians of the participants wrote about their perception of the severity of the social skills deficits exhibited. It was administered to the parents or guardians during each session.

We devised another scale specifically for use in this study, adapted from the Goal Attainment Scaling method (39), and designated it the Homework scale. The Homework scale assesses participant progress on therapeutic goals constructed in collaboration with the participant and their parents, which can be assessed indirectly by quantifying to what extent the participant engaged in therapeutic tasks performed outside the clinical setting, such as conversing with a school peer, or activities of daily living (ADLs), such as tidying their room and bathing. The participant, together with their parents, could select five daily tasks, which were listed on a “homework” sheet and checked off as they were completed. During each subsequent group session, the researcher collected the sheets and tallied the scores.

For the qualitative analysis, four-question interviews were carried out with two former participants (one from each of the two groups) in order to gauge their perception of the group therapy process and the effects of that process beyond the clinical sessions (40).

### 2.3 Materials

The following materials were used: a basic player’s guide book (41); character profiles adapted for use in this study; TTRPG dice kits (containing one four-sided die, one six-sided die, one eight-sided die, one ten-sided die, one 12-sided die, and one 20-sided die); a character and monster miniatures kit. The images were transmitted to a 55-in. television.

### 2.4 Locale

The data were collected in the office of a psychiatrist, in a room decorated with various stimuli from pop culture, containing a square table with eight chairs. During the pandemic, group sessions and the application of some instruments were conducted online (by video conference). For that, we employed the Discord social network, which is a closed platform; that is, only people who have an invitation can enter the server.

### 2.5 Procedure

The first session was in October 2019 and the last was in September 2020. On average, sessions were held once every 2 weeks, excluding the vacation period (mid-December to mid-January) and the period of adaptation for the transition to online sessions at the beginning of the pandemic (March 2020). At the beginning of the pandemic, a return to in-person activities was discussed but did not happen. That is why there was a month-long pause between the last in-person session and the first online session. The data collection timeline was as follows:

➢ The initial application of the IHSA, designated evaluation 1 (Eval. 1), was in the first in-person session.
➢ The next six sessions were conducted normally and in person.
➢ At the end of the sixth session, the IHSA was applied again (Eval. 2).
➢ From the seventh session onward, all sessions were conducted online.
➢ The final application of the IHSA (Eval. 3) was in the last session.

Therefore, the comparison between the first and second applications of the IHSA evaluated the impact of the in-person sessions, and the comparison between the second and third applications evaluated the impact of the online sessions. The comparison between the first and third applications of the IHSA evaluated the overall impact of the intervention.

The group sessions were planned with the aim of intervening in the individual target behavior of each participant. Therefore, although the sessions were held in a group setting, each individual had their own process. Each participant, in collaboration with the therapist, identified their needs, and the therapist planned the reinforcement scheme for that target behavior. Thus, therapeutic objectives were established based on the initial interviews with parents and the participants themselves, always respecting the individual process of each participant and their development. For example, one teenager reported difficulties in conversing and making friends at school. The father provided historical data, stating that his son struggled with making friends at school because he did not engage in conversations or show interest in his peers. The therapist, through discussions and analysis of the needs of that participant, highlighted those difficulties and provided potential paths for successful interaction with others. The teenager expressed challenges in engaging in reciprocal information exchange, and the therapist assisted him in adopting behaviors that could lead to success, such as asking about interests of others and making eye contact. Thus, the intervention always respected the fundamental characteristics of the individual with autism, taking into account signs of discomfort or disappointment. Although the interventions were individualized, they took place simultaneously in group sessions; for example, over the course of a session, the target behavior of one participant was civility and that of another was assertiveness. Target behaviors can be nonverbal, such as eye contact, or verbal, such as empathizing with another participant whose character is harmed during the session. The frequency of training each behavior was determined by opportunity or convenience; rather than having a specific amount of training per session, the frequency could vary depending on the judgment of the primary therapist or co-therapist.

During the online group sessions, the Discord platform was used in order to continue the training of social skills and the sessions were conducted via video conference. However, there were some changes in the training of target behaviors. For example, some target behaviors that were previously addressed in the in-person context, such as eye contact, began to be reinforced when a participant paid attention to other participants. In general, the target social skills continued to be what was important, based on participant complaints, such as behaviors related to civility, engaging in more enjoyable conversations with other participants, empathy, and assertiveness. In addition, in the context of the pandemic, social contact and well-being were primary goals, because it was a very challenging time for autistic participants, given the social isolation. We chose Discord because teenagers identify with that platform and because it is easy to configure bots (robots that perform an automated task) on the platform, which allows server programmers to configure numerous bots that automatically provide reinforcers, with the aim of stimulating socialization. These bots were configured to be online throughout the study period and had functions such as increasing the reinforcing value of the TTRPG, reinforcing social interactions through text and voice channels, and recording the social behaviors displayed. For example, there were some periods during which the server promoted virtual meetings among the group participants. When a participant sent a text message, the bot automatically awarded experience points (XPs), which are required for character evolution, to be added to their character profile in the next group session. There was another bot that helped and reinforced, for example, in recording the duration of conversations on voice channels. To reduce the chances of system failures with bots or imbalance caused by any character having too many XPs, a weekly XP limit was established to prevent any participant from exhibiting excessive screen time behaviors. It is noteworthy that the therapists also participated in social moments outside the group session and engaged in informal conversations with participants, as well as playing online games with them.

As reinforcing stimuli for exhibiting the target behavior, therapists had several options: XP tokens (based on character evolution points); new equipment; a dice bonus; therapist feedback; peer feedback; taking advantage of a facilitating event in the story; a skill bonus; and virtual monetary rewards. The XP tokens could be given during the session, accounted for by the participant, and added to their character profile at the end of the session. In addition, a new sword, armor, shield, or other piece of equipment could be awarded to a participant who exhibited the target behavior. Because the participants were often required to roll a certain number on a die or dice to determine whether a certain effort was successful, a dice bonus (a certain number added to the number rolled) could be granted as an incentive or reinforcement. Furthermore, therapists could compliment and congratulate a participant who exhibited the target behavior, by making a statement such as “Man, you did great in that scene!” The therapists could also encourage the participants to give feedback to each other, or such feedback could be given spontaneously. Typically, when the reinforcer benefited the other team members, they also praised the player involved, making statements such as “Wow, you did great!” and “That helped a lot…” Moreover, when a participant exhibited some appropriate behavior during an event in the story, the therapist could change the direction of the plot to reinforce that behavior, making a statement such as “The Lady of the Potions told you about the fatal weakness of the monster you will face, because you were empathetic with her!” The therapists could also give a skill bonus. For example, the character could gain extra dexterity or extra strength, depending on the situation of the participant. Finally, the participants could earn virtual money within the game system by exhibiting the target response during the session. That money made it possible for their character to acquire utilities.

At approximately 3 years after the final sessions, interviews were conducted with two of the former participants in order to gather qualitative data from the research procedure, with the aim of collecting additional insights not captured through the instruments applied during the study (40). The questions focused on their perception of the use of RPGs for social skills training, their opinions on this approach, the existence of bonds with other participants, and whether any gatherings occurred outside of the clinical context.

### 2.6 Statistical analysis

Continuous variables are expressed as means and standard deviations. The Friedman test, which is a non-parametric test for paired samples and small samples, was used in order to analyze the differences between the three time points, for the total IHSA score and for its six subscales. Values of *p* < 0.05 were considered statistically significant, and a post-hoc test was used in order to determine at which time points there was and was not a significant difference.

Continuous variables are expressed as means and standard deviations. To analyze differences among the three time points, we used the Friedman test, which is a nonparametric paired-sample test. For any result with a value of *p* < 0.05, a post-hoc test was used to determine at which time point there was a significant difference.

## 3 Results

As an initial outcome, the individual characteristics of each research participant were excluded when presenting general information, such as age and gender, along with their clinical condition in Table 1.

**Table 1:** Clinical and demographic characteristics of the participants.

Participant	Diagnosis	Age (years) at baseline	Sex
1	ASD	17	Male
2	ASD	16	Male
3	ASD; ADHD	13	Male
4	ASD	16	Male
5	ASD; ADHD	16	Male
6	ASD; Tourette syndrome	16	Male

ASD, autism spectrum disorder; ADHD, attention-deficit/hyperactivity disorder.

Of the 12 sessions, the first six were conducted in-person and the last six were conducted online. After Eval. 2, the interventions were paused until more information was available about the duration of the quarantine (whether there would be a return to in-person sessions or not). There was also a period of testing in order to adapt the group sessions to an online modality.

After the first six (in-person) sessions, there was an increase in the frequency scores and a decrease in the difficulty scores for specific IHSA domains, and the differences did not reach statistical significance because of the small sample size and because of the change of scenarios from in-person to online. However, there was no significant difference for the total IHSA scores. The mean IHSA scores at all three time points are shown in Table 2.

**Table 2:** Total scores on the Portuguese-language version of the Social Skills Inventory for Adolescents, as well as for its six domains, within the categories frequency and difficulty, among adolescents with autism spectrum disorder undergoing social skills training with a role-playing game, before and during the coronavirus disease 2019 pandemic in Brazil.

Category	Domain	Eval. 1	Eval. 2	Eval. 3	χ^2^	*p*
		Mean ± SD	Mean ± SD	Mean ± SD		
Frequency	All (total score)	86.33 ± 28.23	98.67 ± 25.23	87.33 ± 48.05	1.33	0.51
	Empathy	22.0 ± 9.01	22.5 ± 8.96	18.83 ± 10.61	2.33	0.31
	Self-control	11.0 ± 6.32	16.5 ± 6.32	15.33 ± 9.29	2.33	0.31
	Civility	17.5 ± 6.06	18.83 ± 5.88	16.0 ± 9.08	1.52	0.47
	Assertiveness	13.83 ± 4.45	17.83 ± 5.98	17.17 ± 8.38	2.17	0.34
	Affective perspective	11.17 ± 4.22	12.0 ± 4.82	10.83 ± 7.63	1	0.61
	Social resourcefulness	10.83 ± 4.58	11.0 ± 3.58	9.17 ± 7.52	1.33	0.51
Difficulty	All (total score)	60.0 ± 24.27	38.5 ± 19.31	48.0 ± 33.53	4.96	0.08
	Empathy	16.0 ± 12.31	7.0 ± 4.73	10.17 ± 8.64	1.65	0.44
	Self-control	13.0 ± 4.299	10.83 ± 5.74	14.5 ± 8.69	1.45	0.48
	Civility	9.33 ± 5.2	4.0 ± 3.58	4.33 ± 4.46	2.17	0.34
	Assertiveness	7.67 ± 4.93	5.83 ± 3.6	4.33 ± 4.18	0.09	0.96
	Affective perspective	6.17 ± 2.14	5.5 ± 3.21	8.67 ± 6.09	1.65	0.44
	Social resourcefulness	7.83 ± 4.02	5.33 ± 3.78	6.0 ± 4.73	0.61	0.74

Eval. 1, first evaluation (pre-intervention); Eval. 2, second evaluation (mid-intervention, after six in-person sessions); Eval. 3, third and final evaluation (post-intervention, after six in-person sessions and six online sessions).

Comparing the results obtained at Eval. 1 with those obtained at Eval. 2 (assessing the impact of the in-person sessions), we found that the mean total frequency score on the IHSA increased from 86.33 to 98.67, whereas from Eval. 2 to Eval. 3 (assessing the impact of the online sessions), it decreased to 87.33. From Eval. 1 to Eval. 2, the mean total difficulty score on the IHSA decreased from 60.0 to 38.5. From Eval. 2 to Eval. 3, that score increased to 48.0. For the mean scores for frequency and for difficulty, the standard deviations were greater at Eval. 3, as depicted in Table 2. The same data distribution was observed for the six subscales (IHSA domains).

Analyzing the impact of the in-person sessions, we observed changes in the frequency scores within the various IHSA domains. From Eval. 1 to Eval. 2, the mean frequency score on the empathy subscale increased from 22.0 to 22.5, as did those on the other subscales (Table 2): from 11.0 to 16.5 for self-control; from 17.5 to 18.8 for civility; from 13.8 to 17.8 for assertiveness; from 11.2 to 12.0 for affective perspective; and from 10.8 to 11.0 for social resourcefulness. We also observed changes in the difficulty scores. From Eval. 1 to Eval. 2, the mean difficulty score on the empathy subscale decreased (from 16.0 to 7.0), as did that on the self-control subscale (from 13.0 to 10.8), the civility subscale (from 9.4 to 4.0), the assertiveness subscale (from 7.7 to 5.8), the affective perspective subscale (from 6.2 to 5.5), and the social resourcefulness subscale (from 7.8 to 5.3).

Analyzing the impact of the online sessions, we also observed changes in the frequency scores within the various IHSA domains. From Eval. 2 to Eval. 3, the mean frequency score on all of the subscales increased (Table 2): from 7.0 to 18.8 on the empathy subscale; from 10.8 to 15.3 on the self-control subscale; from 4.0 to 16.0 on the civility subscale; from 5.8 to 17.2 on the assertiveness subscale; from 5.5 to 10.8 on the affective perspective subscale; and from 5.3 to 9.2 on the social resourcefulness subscale.

Over the course of the study, the mean IHSA total frequency score increased from 86.33 (at baseline) to 98.67 (after the six in-person sessions) and then decreased to 87.33 (after the six online sessions). At those same time points, the mean IHSA total difficulty score decreased from 60.0 to 38.5, after which it increased to 48.0. For the mean frequency and difficulty scores, the standard deviations were greater at the post-intervention time point. The mean IHSA total scores and domain scores are shown in greater detail in Table 2.

As can be seen in Table 2, the IHSA scores for all of the frequency domains increased and those for all of the difficulty domains decreased between the first and second evaluations, although the difference was not statistically significant. The frequency score for the self-control domain stands out, because it showed the greatest variation, whereas the difficulty scores showed the greatest variation in the empathy and civility domains. Notably, from the second to the third evaluation, almost all of the scores worsened (frequency scores dropped and difficulty scores rose), the lone exception being the difficulty score for the assertiveness domain.

Because the GCQ assesses participant perception of the behavior of their peers, it was used in order to collect data collected solely from the in-person sessions. The GCQ data collection was suspended by the researcher because of concerns regarding the consistency of the data. Some of the in-person sessions were more tense, with participants exhibiting greater emotional dysregulation and with a greater number of disputes among participants. The mean score on the GCQ engagement subscale ranged from 3.3 to 4.3, whereas that on the avoidance subscale ranged from 1.8 to 3.3 and that on the conflict subscale ranged from 1.7 to 4.0.

All parental responses concerning the severity of and changes in the clinical complaint were analyzed with the CGI scale. According to the data collected, parents highlighted an improvement in the social skills of the participants over the course of the sessions. However the level of parental engagement was low: a total of 54 messages were sent to parents, and only 10 responses were received, translating to a response rate of merely 18.5%.

For the Homework scale, all tasks were tabulated and categorized as ADLs or requests to practice social skills. The distribution between those two categories was 78% for ADLs and 22% for social skills tasks. The mean overall completion rate for these tasks was 78%, regardless of whether the task was related to ADLs or to social skills.

Regarding the qualitative data, the interviews helped us understand the impact of the therapy. Both of the interviewees reported positive experiences with the therapy. Concerning the use of RPGs in therapy, both reported that the RPG was a tool that was fun to use because it mixed real situations with the setting of epic fantasy. They made the following statements:

“… RPG applied to social skills, I thought it was really a super cool thing […] like it was really a way to apply a well-known game to the social environment and I could say that RPG kind of changed my life because it helped me interact more with people.”

“I believe that, in general, the use of RPG as a way to improve social skills was a very useful tool, as it was able to mix real situations with a more playful aspect. Therefore, for me and other people, it was very good, as it made it easier to assimilate and train situations that could happen in our daily lives.”

Another question was about game situations in which social skills, rather than combat, were used as the key solution, despite the fact that combat is a typical characteristic of games of the genre. The interviewees reported the following:

“I remember there was a time when [the therapist/dungeon master] … a character from my team was probably going to die and we were super desperate and didn’t know what to do … we basically had to convince a cleric to heal our character. […] I just wanted to get past that part quickly. […] I had to gather strength [the social skills] to solve this problem.”

“One time that I remember I had to use social skills within the RPG, as my character, was when my character Vandinei, the bard, had to convince Medusa, from Greek mythology, to release us and save other members of the group. And I had to do all this through talking with her. We didn’t enter into any kind of physical fight; my companions and I were injured, and we also had to avoid being turned to stone by her.”

The last two questions were about the friendships made within the therapy groups and the generalization of behaviors beyond the clinical sessions. The former participants made the following statements:

“Yes, I was able to make several friends, from my group and from other RPG groups. I have several friends that I could mention. I would say that [there are] several friends that I made, it was really wonderful. I went to Comic-Con [Electronic gaming event], … I had lunch with a friend of mine and talked about anime [during] the entire lunch … I also went to the cinema several times … I would say that … I made several outings.”

“And I managed to make friendships with group members from that time, I still talk with some of them, not so frequently, but I still maintain a good relationship with them, and the outings that we used to make in groups were mostly going to malls and, especially, to cinemas to watch movies.”

Another important finding of the present study is that the one-year adherence rate was 80%, which is considerably higher that the average rate of 49% reported in comparable clinical studies conducted at other universities, including some in Brazil (42–44).

## 4 Discussion

In this study, we have demonstrated that an RPG-based therapeutic intervention can be used as a means of improving social skills among adolescents with ASD. Although that was our primary objective, the most interesting aspect of our study is probably the abrupt change in the therapeutic setting, which was not initially planned to be online; the pandemic imposed an unexpected change in the intervention. There was, therefore, a period of adaptation to a new technology, with the use of the Discord social platform. Therefore, the impact of the pandemic allowed to draw a comparison between the in-person and online modalities. It behooves us to discuss and analyze the impact of this new technology for use in the online therapeutic setting. After the in-person sessions, there was an increase in the mean frequency scores and a decrease in the mean difficulty scores for appropriate behaviors, although the differences were not significant.

There is some evidence that a growing number of individuals within the ASD community are actually quite comfortable with online interaction (45). In the present study, conducting group sessions on the Discord platform brought numerous qualitative results observed by the experimenters, especially with regard to the generalization of social skills. Participants began to talk more frequently with other participants outside the context of the study, as well as holding numerous virtual meetings during the lockdown, after which they formed some social groups outside the clinical space. The use of a digital platform enabled several interventions that were improved over the initial months of the pandemic. Those interventions are considered to have represented an essential tool for teaching social skills during the study period. We also noticed an increase in social interactions beyond the group sessions (data not shown), which indicates that there is considerable potential for research on the generalization of the social skills acquired in such sessions. For example, we found evidence suggesting that the users spent twice as much time on the server outside of the group sessions than during them. Mainly during 2020, they used Discord to talk about RPGs and other aspects of personal life at different times, rather than only during the clinical sessions.

When comparing the participant adherence rate with the parental response rate (i.e., parental involvement) on the CGI-improvement scale, we found that the latter was relatively low in comparison with the former (18.5% vs. 80%). Although the rate of completion was relatively high (78%) for the tasks evaluated with the Homework scale, there were instances in which the participants reported that they had to remind their parents to check the completion of the tasks and record them on the sheet. This is notable because it suggests high motivation and a commitment to the therapy on the part of the adolescents.

Comparing outcomes between the Homework scale and the CGI scale, we found it interesting that while parents initially seek this type of therapy in the clinic specifically to address social skills deficits, the large majority of the homework tasks chosen, even with parental input, were ADLs rather than tasks related to social skills. This phenomenon could be an interesting subject for future research.

The purpose of utilizing the GCQ was to assess the therapeutic process in a group context. Although the questionnaire was not specifically formulated to measure social skills, it at least provided insights into the process and climate of each group session. For instance, the data obtained from the groups indicated that there was instability in some sessions. Perhaps future research could lead to the development of an instrument capable of evaluating the evolution of the repertoire of social skills exhibited in group therapy sessions.

The effects of the pandemic and the impact of the use of online platforms initially imposed certain barriers to social skills training, such as the absence of physical contact, as well as the inability to distinguish facial expressions, discriminate nuances in tone of voice, or identify other paralinguistic components. However, online social skills training cannot be completely discounted. This could have opened a vast new area of study, because the process of adapting the interventions to an online modality made it necessary for us to learn how to use some digital tools that can be very useful for the study of the generalization of social skills. As in the PEERS protocol, the use of digital tools and social networks is one step in our social skills training protocol. The Discord platform allowed a variety of interventions.

In the study conducted by Katō (22), who used an RPG as a therapeutic tool, there were gains in terms of motivation. However, in the present study, the proposal was to use knowledge in the field of social skills to establish domains of behavior, as described by Del Prette and Del Prette (3) and, through functional analysis, to apply an individualized, appropriate intervention; that is, to use the elements of an RPG and gamification to evoke and enhance complex social skills. Therefore, reconciling the area of RPGs, other games, gamification, therapy, and the field of social skills requires technical know-how and up-to-date knowledge of the clinical aspects. In the fusion between these areas, it is important to take care to keep technical knowledge about mental health and clinical conditions up to date, so that it is not secondary to the study or knowledge of the RPG system.

### 4.1 Limitations

Our study has some limitations. The small sample size and number of sessions limited the power of the study to detect significant differences, such as those between the in-person and online sessions in terms of the frequency scores and difficulty scores for appropriate behaviors. Therefore, further studies are needed in order to evaluate those variables with greater precision. Although the pandemic had a relevant impact, the worsening of the scores for practically all of the IHSA domains could lead to the conclusion that the online modality for social skills training represented a limitation. However, it is possible that the IHSA is not the appropriate tool for evaluating a virtual context. We chose to use the IHSA because it has been employed in various studies in the scientific literature of Brazil. In the international context, other inventories, such as the Social Skills Rating Scale, are employed. However, both instruments are of the pre- and post-test evaluation type. Although the IHSA is a self-report questionnaire, the instruction given to the subject is hypothetical and deductive. For each item, the subject is asked to provide an answer based on the last ten situations, stating how many times they behaved appropriately and, if not, imagining what they should have done in each situation. Despite the fact that the IHSA has been validated for use in adolescents, its application in the present study is questionable, given that, when the participants were isolated by the pandemic, they did not experience the natural conditions described in the items on the IHSA. Therefore, the IHSA might not be the best tool for assessing the social repertoire of individuals during a period of little or no social exposure (as during quarantine). In addition, some participants reported that it made them uncomfortable and, in their words, “had nothing to do” with the specific items regarding condom use or talking to parents about sex. Some participants told the therapists, “I do not know how to answer this question; it never crosses my mind.” Another item that was criticized was “showing annoyance if your brother/sister messes with your things.” Many of the participants did not have siblings, so asking them to imagine a sibling does not seem like a good way to evaluate social skills. Therefore, either the IHSA needs to be revised or a new instrument should be developed for this purpose. One final limitation is that we did not apply any instrument to assess quality of life, which could have provided valuable information. Nevertheless, it is important to note that the social skills training proposed in this work is not the traditional form, which has been criticized (9), because the therapeutic objectives are aligned with the preferences of the individuals with autism and the receptivity of other members of the therapy group.

## 5 Conclusion

In conclusion, the field of social skills continues to develop. Future studies should employ quality of life inventories and qualitative data, because social skills continue to be relevant to most human experiences. Such studies could investigate the effects that combining RPGs and psychotherapy, as a method of social skills training, has on the well-being of the participants.

## Data availability statement

The original contributions presented in the study are included in the article/supplementary materials, further inquiries can be directed to the corresponding author.

## Ethics statement

The studies involving humans were approved by Comitê de Ética do Instituto de Psicologia da Universidade de São Paulo. The studies were conducted in accordance with the local legislation and institutional requirements. Written informed consent for participation in this study was provided by the participants’ legal guardians/next of kin.

## Author contributions

GH: Conceptualization, Data curation, Formal analysis, Investigation, Methodology, Writing – original draft. RO: Methodology, Writing – review & editing. MA: Methodology, Writing – review & editing. ReG: Methodology, Writing – review & editing. RB: Methodology, Writing – review & editing. RoG: Methodology, Writing – review & editing. LF: Methodology, Writing – review & editing. AL: Methodology, Writing – review & editing. MO: Methodology, Writing – review & editing. DO: Methodology, Writing – review & editing. MW: Methodology, Writing – review & editing. LM: Methodology, Writing – review & editing. FN: Conceptualization, Formal analysis, Investigation, Methodology, Supervision, Visualization, Writing – review & editing.
